# Genome sequence of two novel virulent clinical strains of *Burkholderia pseudomallei* isolated from acute melioidosis cases imported to Israel from India and Thailand

**DOI:** 10.1186/s12863-024-01225-x

**Published:** 2024-05-23

**Authors:** Inbar Cohen-Gihon, Galia Zaide, Sharon Amit, Iris Zohar, Orna Schwartz, Yasmin Maor, Ofir Israeli, Gal Bilinsky, Ma’ayan Israeli, Shirley Lazar, David Gur, Moshe Aftalion, Anat Zvi, Adi Beth-Din, Erez Bar-Haim, Uri Elia, Ofer Cohen, Emanuelle Mamroud, Theodor Chitlaru

**Affiliations:** 1 Department of Biochemistry and Molecular Genetics, Institute for Biological Research, Ness-Ziona, Israel; 2 Clinical Microbiology, Sheba Medical Center, Tel Hashomer, Israel; 3 Faculty of Medicine, Tel-Aviv University, Tel-Aviv, Israel; 4 Infectious Disease Unit, Wolfson Medical Center, Holon, Israel; 5 Microbiology and Immunology Laboratory Wolfson Medical Center, Holon, Israel; 6 Faculty of Digital Technologies in Medicine, Holon Institute of Technology, Holon, Israel

**Keywords:** *Burkholderia pseudomallei*, Melioidosis, Genome sequence, Virulence genes, Phylogenetic tree

## Abstract

**Objective:**

*Burkholderia pseudomallei*, the etiological cause of melioidosis, is a soil saprophyte endemic in South-East Asia, where it constitutes a public health concern of high-priority. Melioidosis cases are sporadically identified in nonendemic areas, usually associated with travelers or import of goods from endemic regions. Due to extensive intercontinental traveling and the anticipated climate change-associated alterations of the soil bacterial flora, there is an increasing concern for inadvertent establishment of novel endemic areas, which may expand the global burden of melioidosis. Rapid diagnosis, isolation and characterization of *B. pseudomallei* isolates is therefore of utmost importance particularly in non-endemic locations.

**Data description:**

We report the genome sequences of two novel clinical isolates (MWH2021 and MST2022) of *B. pseudomallei* identified in distinct acute cases of melioidosis diagnosed in two individuals arriving to Israel from India and Thailand, respectively. The data includes preliminary genetic analysis of the genomes determining their phylogenetic classification in rapport to the genomes of 131 *B. pseudomallei* strains documented in the NCBI database. Inspection of the genomic data revealed the presence or absence of loci encoding for several documented virulence determinants involved in the molecular pathogenesis of melioidosis. Virulence analysis in murine models of acute or chronic melioidosis established that both strains belong to the highly virulent class of *B. pseudomalleii*.

**Supplementary Information:**

The online version contains supplementary material available at 10.1186/s12863-024-01225-x.

## Objective

The Gram-negative bacterial pathogen *Burkholderia pseudomallei* is the etiological cause of melioidosis. As of 2018 there were 165,000 global estimated annual morbidities, including 89,000 deaths, mostly in Southeast Asia where the disease is endemic [1]. *B. pseudomallei* is considered a high priority biohazard due to its prevalence in soil and water, high virulence associated with inhalation exposure, low infective dose, high mortality rates, native resistance to a wide range of antibiotics, and paucity of an efficient licensed vaccine [2–4]. Melioidosis symptoms are nonspecific, thus hindering identification of the disease, which may inadvertently be diagnosed as tuberculosis or a common form of pneumonia [1, 5, 6]. The *B. pseudomallei* genome is highly plastic, resulting in significant sequence variability amongst strains [7]. Melioidosis cases are sporadically identified in nonendemic areas, usually associated with travelers or transport of goods from endemic regions. Due to extensive intercontinental traveling and the anticipated climate change-associated alterations of the soil bacterial flora, there is an increasing concern for inadvertent establishment of novel endemic areas which may expand the global burden of melioidosis. Rapid diagnosis, isolation and characterization of *B. pseudomallei* isolates is therefore of utmost importance, in particular in non-endemic locations.

Two Israelis returning from Southeast Asia, one from Thailand and the other from India, were hospitalized in different medical centers due to fulminant symptoms reminiscent of acute pneumonia, subsequently diagnosed as travel-related melioidosis. The objective of the study documented in this brief data note, was genetic identification of the etiologic pathogen isolated from these clinical cases, and analysis of the respective bacterial genomes for taxonomic typing and primary determination of their genetic characteristics.

## Data description

We report the draft genome of two novel clinical *B. pseudomallei* strains denoted MWH2021 and MST2022 isolated from melioidosis cases diagnosed in Israel, associated with travelers from India and Thailand, respectively.

Previously, three additional clinical isolates of melioidosis were documented in Israel, from Thailand [8], Eritrea [9] and India [10, 11]. The strains described in the current report were acknowledged as *B. pseudomallei* by the Vitek KL2 bacterial identification system and MALDI-TOF (Bruker) mass spectrometric analysis and confirmed by PCR analysis [12]. Virulence analysis of the strains was performed in the melioidosis murine model [13, 14]. MWH2021 strain exhibited an intranasal (IN) Lethal Dose 50% (LD_50_; more than 30 days survival of mice inoculated with increasing doses, calculated by linear regression using the GraphPad Prism V5 software [10, 13, 15]) of 126 CFU and 950 CFU in the BALB/c and C57BL/6J strains of mice, respectively. LD_50_ of strain MST2022 was 13 CFU and 83 CFU (BALB/c and C57BL/6J mice, respectively). Of note, Balb/c serves for modeling the acute form of melioidosis while C57BL/6J recapitulates the chronic form. The data indicate that both novel clinical isolates belong to the group of highly virulent strains [16].

Genome sequencing *of B. pseudomallei* MWH2021 and MST2022 isolates employed purified chromosomal DNA isolated from BHI-agar colonies, which served for generation of genomic libraries (Nextera XT kit, Illumina). Sequencing of both strains was performed on Miseq Instrument (Illumina), generating short-read sequences. Long-read sequencing using Oxford Nanopore Technologies, produced additional data sets which enabled improved assembly [13].

The genomic sequences of the two novel strains were deposited to the NCBI database [17–20]. The data deposited in the various public databases as well as supplementary material pertaining to this data note are detailed in Table 1. To determine the phylogenetic relation of the novel strains to other *B. pseudomallei* strains. A total of 131 complete genome sequences were downloaded from NCBI [21]. Core genome alignment and phylogeny of the strains relative to the reference genome strain Mahidol-1106a (accession number GCF_000756125.1) were performed using the Parsnp software, v1.2 [22]. The resulting phylogenetic tree, depicted in the Supplementary Fig. 1 [23] was shaped with the iTOL: interactive tree of life platform [24]. This analysis established that the novel MWH2021 strain is adjacent in the phylogenetic clustering to strain MAA2018 [10, 11], in line with their common geographic origin (India). The two isolates differ by 8,879 and 7,408 SNPs on chromosome 1 and 2, respectively, clearly indicating that in spite of their phylogenetic vicinity they represent distinct strains. In general, as expected, the analysis established a strong correlation between genomic DNA sequence similarities of various strains and their respective geographical origin (see Supplementary Fig. 1).

The genomes of the novel strains, as well as those of the three additional strains BP1, BP2 and MAA2018 previously isolated and documented in Israel from cases of melioidosis [8–11, 13], were interrogated for the presence of 36 genes encoding for potential *B. pseudomallei* virulence factors [1, 3]. The results summarized in Supplementary Data 2 [14], show that all these genes are present in the genome of the novel strain MWH2021. In this regard, this strain does not differ from the previously documented strain of Indian origin, in line with their phylogenetic proximity. Three genes (*chbP*, *boaB* and *boaA*) were not identified in the genome of the novel strain MST2022. While these genes encode for factors believed to be involved in the virulence of the bacteria [25–27], their absence did not correlate with decreased virulence of the MST2022 [14], in accordance with the notion that pathology of melioidosis involves the activity of numerous bacterial factors whose individual contribution to virulence is difficult to assess.

**Table 1:** Overview of data files/data sets

Label	Name of data file/data set	File types (file extension)	Data repository and identifier (DOI or accession number)	Ref. (Repository)
Data set 1	*B. pseudomallei* MWH2021 assembly	DNA sequence (.fasta)	https://identifiers.org/ncbi/insdc.gca:GCA_030913145.1	17 (NCBI)
Data set 2	*B. pseudomallei* MST2022 assembly	DNA sequence (.fasta)	http://identifiers.org/insdc.gca:GCA_030144945.1	18 (NCBI)
Data set 3	*B. pseudomallei* MWH2021 raw reads	DNA sequence (.fastq)	https://identifiers.org/ncbi/insdc.sra:SRP483724	19 (NCBI)
Data set 4	*B. pseudomallei* MST2022 raw reads	DNA sequence (.fastq)	https://identifiers.org/ncbi/insdc.sra:SRP483736	20 (NCBI)
Supplementary Fig. 1	Phylogenetic tree of *B. pseudomallei* strains	Microsoft Power Point (.pptx)	10.6084/m9.figshare.24961800	23 (Figshare)
Supplementary Data 1	Supplementary Method Data	MicrosoftWord (.docx)	10.6084/m9.figshare.25204352	13 (Figshare)
Supplementary Data 2	LD_50_ values and virulence determinants	MicrosoftWord (.docx)	10.6084/m9.figshare.25204568	14 (Figshare)
Supplementary Table 1	NCBI *BP* complete genomes as of June 2023	Excel (.xls)	10.6084/m9.figshare.25479535	28 (Figshare)

## Limitation

Bioinformatic DNA similarity analysis of the genomic sequences of the two novel clinical isolates, for determining their phylogenetic relation to other *B. pseudomallei* strains [23, 28], was conducted using 131 complete *B. pseudomallei* genomes present in the NCBI databank (as of June 2023). It is conceivable that conducting the analysis by comparison to all available *B. pseudomallei* genomes (not only complete ones), may have provided a more comprehensive phylogenetic profile.

The bioinformatics screen of the sequences for potential virulence factors did not include inspection of the possible point mutations or indels (insertions/deletions) but only presence or absence of the respective orthologous genes, therefore the study cannot attest for their level of expression.

### Electronic supplementary material

Below is the link to the electronic supplementary material.

Supplementary Material 1

Supplementary Material 2

Supplementary Material 3

Supplementary Material 4

## Data Availability

The data described in this Data note can be freely and openly accessed on NCBI under [GCF_030913145.1; GCA_030144945.1; PRJNA1008066; PRJNA962450]. Please see Table 1 and references [13, 14, 17-20, 23, 28] for details and links to the data.

## Abbreviations

*B. pseudomallei*: *Burkholderia pseudomallei*
MWH2021: **M**elioidosis case from **W**olfson Hospital, **H**olon, **2021**
MST2022: **M**elioidosis case from **Sheba** Hospital, **T**el-Hashomer, **2022**
