# Supplementary material for: Genome sequence of two novel virulent clinical strains of Burkholderia pseudomallei isolated from acute melioidosis cases imported to Israel from India and Thailand
Source: BMC Genom Data. 2024 May 23;25:47. doi: 10.1186/s12863-024-01225-x (PMC11118722; doi:10.1186/s12863-024-01225-x)
Supplement: Supplementary file 1 — Supplementary Material 1 [file 12863_2024_1225_MOESM1_ESM.docx]

**Supplementary Method Data**

**Methodology details pertaining to publication:**

[**Genome Sequence of**](https://mra.asm.org/content/7/11/e01049-18) **Two Novel Virulent Clinical Strains of *Burkholderia pseudomallei* Isolated from Acute Melioidosis Cases Imported to Israel from India and Thailand**

Inbar Cohen-Gihon^a^, Galia Zaide^a^, Sharon Amit^b,c^, Iris Zohar^c,d^, Orna Schwartz^e^, Yasmin Maor^c,d^, Ofir Israeli^a^, Gal Bilinsky^a^, Ma’ayan Israeli^a^, Shirley Lazar^a^, David Gur^a^, Moshe Aftalion^a^, Anat Zvi^a^, Adi Beth-Din^a^, Erez Bar-Haim^a^, Uri Elia^a^, Ofer Cohen, Emanuelle Mamroud^a^ and Theodor Chitlaru^a,f*^

^a^ Department of Biochemistry and Molecular Genetics, Israel Institute for Biological Research, Ness-Ziona, Israel

^b^ Clinical Microbiology Laboratory Sheba Medical Center, Tel Hashomer, Israel

^c^ Faculty of Medical and Health Sciences, Tel-Aviv University, Tel-Aviv, Israel

^d^ Infectious Disease Unit, Edith Wolfson Medical Center, Holon, Israel

^e^ Microbiology and Immunology Laboratory Wolfson Medical Center, Holon, Israel

^f^ Faculty of Digital Technologies in Medicine, Holon Institute of Technology, Holon, Israel

1. **Details regarding *Burkholderia pseudomallei* strains BP1, BP2 and MAA2018**

**Strains**: *B. pseudomallei* strains used in the study are: BP1, BP2, MAA2018, **MWH2021, and MST2022**. BP1 and BP2 strains originated from patients traveling from Thailand and Eritrea, respectivly. The genome of the BP1 clinical isolate (described by Cahn et al., see reference 1 below) exhibits some homology to the 1710b strain (NCBI accession numbers NC_007434 and NC_007435) yet it differs significantly from this strain by 11259 and 10865 SNPs on chromosome 1 and 2, respectively (ribosomal RNA sequences of the BP1 strain are available in the NCBI database as strain Had_B73694, accession number FJ426359.1). BP2 exhibits homology to the 1106a strain (NCBI accession numbers NC_009076 and NC_009078) yet it differs from this strain by 1568 and 1436 SNPs on chromosome 1 and 2, respectively. The strain MAA2018 is extensively detailed in references 2 and 3. The genome sequence of strain MAA2018 is available in the NCBI data base as Projects [SLUE01000001](https://www.ncbi.nlm.nih.gov/nuccore/SLUE01000001) to [SLUE01000183](https://www.ncbi.nlm.nih.gov/nuccore/SLUE01000183) (BioProject [PRJNA525961](https://www.ncbi.nlm.nih.gov/bioproject/PRJNA525961) and BioSample number [SAMN11081001](https://www.ncbi.nlm.nih.gov/biosample/SAMN11081001)). Handling of live *B. pseudomallei* was under BL3 laboratory safety conditions. The strains **MWH2021, MST2022** are described in the Data Note to which the current Methodology document pertains.

1. **Details regarding virulence experimentation of *Burkholderia pseudomallei* strains BP1, BP2, MAA2018, MWH2021 and MST2022**

**Animal experimentation:** BALB/c (20-25 g, Charles River, UK) or C57BL/6J (20-25 g, Jackson) were infected for virulence evaluation. For infection, bacterial cultures were set in LB media by inoculation with an over-night starter at an initial density of 0.05xOD units. Bacteria were grown for 3-4 hours at 37^o^C, to mid-logarithmic phase, (approx. 2xOD units), centrifuged and re-suspended in PBS at the desired concentration (1xOD unit=5x10^8^ CFU) such that mice were infected intranasally (IN) with 40 μl or subcutaneously (SC) with 100 μl bacterial suspension and serial 10-fold dilutions. A total of 5 mice per dose were used (range 10-10^6^ for IN infection and 10^3^-10^8^ for SC infection). The remaining bacterial dose suspensions were plated for total viable counts (CFU ml^-1^) to confirm the dose administered to the animals. The animals were observed daily for 30 days. The lethal dose required to kill 50% (LD_50_) of the animals was calculated by non-linear fit regression using the GraphPad Prism (version 5.0) statistical analysis software (San Diego, CA). Animal experiments were approved by the Israeli Institute for Biological Research (IIBR) committee for animal research. The IIBR animal-experiment protocol numbers were M-41-18 and M-13-22. The experimental animals were handled according to the National Research Council 1996 Guide for the Care and Use of Laboratory Animals and regulations of the IIBR Animal Use Committee.

1. **Details regarding genome sequencing and bioinformatics phylogeny analysis of *Burkholderia pseudomallei* strains**

For determining the genome sequence of the novel *B. pseudomallei* MWH2021 and MST2022 clinical isolates, DNA was isolated from colonies cultivated for 48 hours on Brain Heart Infusion Agar (BHI), suspended in 1 mL PBS, combined at a 1:1 ratio with ATL buffer (Quigen), and subjected to heat inactivation and lysis by heating at 100°C for 30 minutes. Subsequently, the DNA was purified using the QIAamp DNA blood minikit ( Qiagen). A quantity of 1 ng of DNA was extracted from each sample to create genomic libraries, employing the Nextera XT kit by Illumina. Short-read sequencing of the entire genome in paired-end (PE) mode was performed on a MiSeq instrument using the 300-PE v2 kit from Illumina. Adapter sequences were removed using Trim Galore v0.6.6 [4] with default parameters. The resulting Illumina data sets contained 7,818,642 read pairs for strain MWH2021 and 4,637,571 read pairs for strain MST2022.

For long-read sequencing using Oxford Nanopore Technologies (ONT), libraries were constructed following the manufacturer protocols using the Ligation Sequencing Kit (SQK-LSK109) along with NEBNext® Companion Module (E7180L) and Native Barcode Kits (EXP-NBD104, EXP-NBD114). All experiments were conducted on Nanopore R9.4.1 flow cells and the MinION Mk1B device. Following sequencing, Guppy (v5.0.16) software was utilized for high accuracy basecalling (HAC) and demultiplexing [5]. The final long-read data sets contained 662,928 reads at an average length of 651 bp for strain MWH2021 and 754,131 reads at an average length of 680 bp for strain MST2022.

Genome sequences assembly for the two novel strains were generated by combining data from both the Illumina and MinION data sets using Unicycler v0.4.8 [6], using the following parameters: -t 48, --linear_seqs 2. The assembled genome of MWH2021 consists of 7,600,474 bp with a GC content of 67.4% and N50 of 223,403 nt. The assembled genome of MST2022 consists of 7,118,052 bp with a GC content of 67.7% and N50 of 299,719 nt.

The genomic sequences of the two novel strains were deposited to the NCBI database as detailed in Table 1. To determine the phylogenetic relation of the novel strains to other *B. pseudomallei* strains, complete *B. pseudomallei* genomic assemblies were downloaded from NCBI [7], resulting in a total of 131 genomes (as of June 2023). Core genome alignment and phylogeny of the assemblies were performed using the Parsnp software [8] v1.2 with respect to the reference genome *B. pseudomallei* strain Mahidol-1106a (GCF_000756125.1), using the following parameters: -aligner libMUSCLE -threads 60. In doing so, this utility extracts the core SNP information derived from the alignments, providing the genetic variation insights.

1. **Details regarding genome bioinformatics survey of loci encoding for potential virulence factors in the genomes *B. pseudomalle*i strains BP1, BP2, MAA2018, MWH2021 and MST2022**

Identification of BP1, BP2, MAA2018, MWH2021 and MST2022 orthologous sequences to 36 genes previously documented as virulence factors (VF) in various *B. pseudomallei* strains [9, 10] was conducted. The sequences of these 36 genes were retrieved from the reference *B. pseudomallei* ASM75612V1 genome, and sequence similarity search against the genomic contigs of BP1, BP2, MAA2018, MWH2021 and MST2022 was performed by Blast analysis [11]. Standard definition of orthologs (expectation value of e^-10^ or lower and a coverage above 80%) was implemented. To avoid false negative results which may result from the discontinuous nature of the contigs, a complimentary strategy was applied to further assess the presence of the VFs documented genes in the genomes of the 5 strains. To this end, the extent of coverage obtained by the resequencing procedure at the VF genes location in the reference genome was explicitly verified.

1. **References**
2. Cahn A, Koslowsky B, Nir-Paz R, Temper V, Hiller N, Karlinsky A, Gur I, Hidalgo-Grass C, Heyman SN, Moses AE, Block C. 2009. Imported melioidosis, Israel, 2008. Emerg Infect Dis 15:1809–1811. doi: 10.3201/eid1511.090038.
3. Brosh-Nissimov T, Grupel D, Abuhasira S, Leskes H, Israeli M, Lazar S, Elia U, Israeli O, Beth-Din A, Bar-Haim E, Cohen-Gihon I, Zvi A, Cohen O, Chitlaru T. Am J Trop Med Hyg. 2019 Sep;101(3):580-584. doi: 10.4269/ajtmh.19-0303. PMID: 31287043.
4. Israeli O, Cohen-Gihon I, Brosh-Nissimov T, Zvi A, Beth-Din A, Shifman O, Israeli M, Elia U, Lazar S, Bar-Haim E, Cohen O, Chitlaru T. Microbiol Resour Announc. 2019 May 9;8(19):e00281-19. doi: 10.1128/MRA.00281-19. PMID: 31072902.
5. Trim Galore is a wrapper script to automate quality and adapter trimming as well as quality control <https://github.com/FelixKrueger/TrimGalore>.
6. Analysis solutions for nanopore sequencing data. Nanopore sequencing data analysis https://nanoporetech.com.
7. Wick RR, Judd LM, Gorrie CL, Holt KE. Unicycler: Resolving bacterial genome assemblies from short and long sequencing reads. PLoS Comput Biol. 2017 Jun 8;13(6):e1005595. doi: 10.1371/journal.pcbi.1005595. PMID: 28594827
8. <https://www.ncbi.nlm.nih.gov/datasets/genome/?taxon=28450>
9. Treangen TJ, Ondov BD, Koren S, Phillippy AM. The Harvest suite for rapid core-genome alignment and visualization of thousands of intraspecific microbial genomes. Genome Biol. 2014;15(11):524.
10. Wiersinga WJ, Virk HS, Torres AG, Currie BJ, Peacock SJ, Dance DAB, Limmathurotsakul D. Melioidosis. Nat Rev Dis Primers. 2018 Feb 1;4:17107. doi: 10.1038/nrdp.2017.107. PMID: 29388572; PMCID: PMC6456913.
11. Meumann EM, Limmathurotsakul D, Dunachie SJ, Wiersinga WJ, Currie BJ. Burkholderia pseudomallei and melioidosis. Nat Rev Microbiol. 2023 Oct 4. doi: 10.1038/s41579-023-00972-5. Epub ahead of print. PMID: 37794173.
12. Altschul, S.F., Gish, W., Miller, W., Myers, E.W., Lipman, D.J. (1990) “Basic local alignment search tool.” J. Mol. Biol. 215:403-410.
