# Supplementary material for: Genome sequence of two novel virulent clinical strains of Burkholderia pseudomallei isolated from acute melioidosis cases imported to Israel from India and Thailand
Source: BMC Genom Data. 2024 May 23;25:47. doi: 10.1186/s12863-024-01225-x (PMC11118722; doi:10.1186/s12863-024-01225-x)
Supplement: Supplementary file 2 — Supplementary Material 2 [file 12863_2024_1225_MOESM2_ESM.docx]

**Supplementary Data**

**LD50 values and virulence determinants**

**Experimental LD50 values and virulence determinants of *Burkholderia pseudomallei* strains**

Supplementary information pertaining to publication

[**Genome Sequence of**](https://mra.asm.org/content/7/11/e01049-18) **Two Novel Virulent Clinical Strains of *Burkholderia pseudomallei* Isolated from Acute Melioidosis Cases Imported to Israel from India and Thailand**

Inbar Cohen-Gihon^a^, Galia Zaide^a^, Sharon Amit^b,c^, Iris Zohar^c,d^, Orna Schwartz^e^, Yasmin Maor^c,d^, Ofir Israeli^a^, Gal Bilinsky^a^, Ma’ayan Israeli^a^, Shirley Lazar^a^, David Gur^a^, Moshe Aftalion^a^, Anat Zvi^a^, Adi Beth-Din^a^, Erez Bar-Haim^a^, Uri Elia^a^, Ofer Cohen, Emanuelle Mamroud^a^ and Theodor Chitlaru^a,f*^

^a^ Department of Biochemistry and Molecular Genetics, Israel Institute for Biological Research, Ness-Ziona, Israel

^b^ Clinical Microbiology Laboratory Sheba Medical Center, Tel Hashomer, Israel

^c^ Sackler Faculty of Medicine, Tel-Aviv University, Tel-Aviv, Israel

^d^ Infectious Disease Unit, Wolfson Medical Center, Holon, Israel

^e^ Microbiology and Immunology Laboratory Wolfson Medical Center, Holon, Israel

^f^ Faculty of Digital Technologies in Medicine, Holon Institute of Technology, Holon, Israel

**A: Murine Intra-nasal Lethal Dose 50% (LD50_IN_) values (CFU/mouse) experimentally determined for *Burkholderia pseudomallei* strains in BALB/c and C57BL/6J mice as described in Supplementary Method Data (https://figshare.com/search?q=10.6084%2Fm9.figshare.25204352)**

|  |  |  |  | **Novel Strains** | |
| --- | --- | --- | --- | --- | --- |
| **Murine Strain** | **BP1** | **BP2** | **MAA2018** | **MWH2021** | **MST2022** |
| **BALB/c** | **1** | **2** | **6** | **126** | **13** |
| **C57BL/6J** | **140** | **60** | **30** | **950** | **83** |

**B**: **Genomic sequences of the *Burkholderia pseudomallei* strains were analyzed for determining the presence (+) or absence (-) of 36 potential virulence factors (reviewed in references 1 and 2), as described in Supplementary Method Data (https://figshare.com/search?q=10.6084%2Fm9.figshare.25204352)**

1. Wiersinga, W. J, Virk H.S., Torres, A.G., Currie, B.J., Peacock, S.J., Dance, D.A.B., Limmathurotsakul, D. 2018. Melioidosis. Nat Rev Dis Primers 4:17107. doi: 10.1038/nrdp.2017.107

2. Limmathurotsakul, D., Golding, N., Dance, D.A., Messina, J.P., Pigott, D.M., Moyes, C.L., Rolim, D.B., Bertherat, E., Day, N.P., Peacock, S.J., Hay, S.I. 2016. Predicted global distribution of *Burkholderia pseudomallei* and burden of melioidosis. Nat Microbiol 11:15008. doi: 10.1038/nmicrobiol.2015

|  | **Gene Name** | **Locus Tag** | **Accession No.** | **BP1** | **BP2** | **MAA2018** | **MWH2021** | **MST2022** |
| --- | --- | --- | --- | --- | --- | --- | --- | --- |
| 1 | chbP | BPSS1385 | WP_009934501.1 | **-** | **-** | **+** | **+** | **-** |
| 2 | sodC | DP58_RS03040 | [WP_004522548.1](https://www.ncbi.nlm.nih.gov/protein/WP_004522548.1) | **+** | **+** | **+** | **+** | **+** |
| 3 | ispH | DP58_RS03395 | [WP_004535362.1](https://www.ncbi.nlm.nih.gov/protein/WP_004535362.1) | **+** | **+** | **+** | **+** | **+** |
| 4 | purN | DP58_RS03450 | [WP_004522603.1](https://www.ncbi.nlm.nih.gov/protein/WP_004522603.1) | **+** | **+** | **+** | **+** | **+** |
| 5 | waaF | DP58_RS04090 | [WP_004526219.1](https://www.ncbi.nlm.nih.gov/protein/WP_004526219.1) | **+** | **+** | **+** | **+** | **+** |
| 6 | pilA | DP58_RS04135 | [WP_004526211.1](https://www.ncbi.nlm.nih.gov/protein/WP_004526211.1) | **+** | **+** | **+** | **+** | **+** |
| 7 | boaB | BPS_RS09025 | [WP_011325557.1](https://www.ncbi.nlm.nih.gov/protein/WP_011325557.1) | **-** | **-** | **+** | **+** | **-** |
| 8 | tssM | DP58_RS09700 | [WP_004531876.1](https://www.ncbi.nlm.nih.gov/protein/WP_004531876.1) | **+** | **+** | **+** | **+** | **+** |
| 9 | katG | DP58_RS10970 | [WP_004194237.1](https://www.ncbi.nlm.nih.gov/protein/WP_004194237.1) | **+** | **+** | **+** | **+** | **+** |
| 10 | dpsA | DP58_RS10980 | [WP_004200519.1](https://www.ncbi.nlm.nih.gov/protein/WP_004200519.1) | **+** | **+** | **+** | **+** | **+** |
| 11 | purM | DP58_RS11210 | [WP_004194386.1](https://www.ncbi.nlm.nih.gov/protein/WP_004194386.1) | **+** | **+** | **+** | **+** | **+** |
| 12 | rpoE | DP58_RS13135 | NA | **+** | **+** | **+** | **+** | **+** |
| 13 | wcbT | BPS_RS14975 | [WP_004194209.1](https://www.ncbi.nlm.nih.gov/protein/WP_004194209.1) | **+** | **+** | **+** | **+** | **+** |
| 14 | wcbS | BPS_RS14980 | [WP_004527661.1](https://www.ncbi.nlm.nih.gov/protein/WP_004527661.1) | **+** | **+** | **+** | **+** | **+** |
| 15 | wcbR | BPS_RS14985 | [WP_004550042.1](https://www.ncbi.nlm.nih.gov/protein/WP_004550042.1) | **+** | **+** | **+** | **+** | **+** |
| 16 | luxR | DP58_RS15110 | [WP_004547256.1](https://www.ncbi.nlm.nih.gov/protein/WP_004547256.1) | **+** | **+** | **+** | **+** | **+** |
| 17 | rpoS | DP58_RS15680 | [WP_004193640.1](https://www.ncbi.nlm.nih.gov/protein/WP_004193640.1) | **+** | **+** | **+** | **+** | **+** |
| 18 | blf1 | DP58_RS15910 | [WP_004531382.1](https://www.ncbi.nlm.nih.gov/protein/WP_004531382.1) | **+** | **+** | **+** | **+** | **+** |
| 19 | fliC | BPS_RS17885 | [WP_004198207.1](https://www.ncbi.nlm.nih.gov/protein/WP_004198207.1) | **+** | **+** | **+** | **+** | **+** |
| 20 | bsaQ | DP58_RS20125 | [WP_004188490.1](https://www.ncbi.nlm.nih.gov/protein/WP_004188490.1) | **+** | **+** | **+** | **+** | **+** |
| 21 | bsaU | DP58_RS20145 | [WP_004536823.1](https://www.ncbi.nlm.nih.gov/protein/WP_004536823.1) | **+** | **+** | **+** | **+** | **+** |
| 22 | bsaZ | DP58_RS20170 | [WP_004533159.1](https://www.ncbi.nlm.nih.gov/protein/WP_004533159.1) | **+** | **+** | **+** | **+** | **+** |
| 23 | bipB | DP58_RS20180 | [WP_004537550.1](https://www.ncbi.nlm.nih.gov/protein/WP_004537550.1) | **+** | **+** | **+** | **+** | **+** |
| 24 | bipC | DP58_RS20185 | [WP_004551891.1](https://www.ncbi.nlm.nih.gov/protein/WP_004551891.1) | **+** | **+** | **+** | **+** | **+** |
| 25 | bipD | DP58_RS20195 | WP_004537374.1 | **+** | **+** | **+** | **+** | **+** |
| 26 | bopE | DP58_RS20215 | [WP_004188462.1](https://www.ncbi.nlm.nih.gov/protein/WP_004188462.1) | **+** | **+** | **+** | **+** | **+** |
| 27 | bopA | DP58_RS20225 | [WP_004536698.1](https://www.ncbi.nlm.nih.gov/protein/WP_004536698.1) | **+** | **+** | **+** | **+** | **+** |
| 28 | vgrG | DP58_RS20325 | [WP_004533112.1](https://www.ncbi.nlm.nih.gov/protein/WP_004533112.1) | **+** | **+** | **+** | **+** | **+** |
| 29 | hcp1 | DP58_RS20350 | [WP_004525344.1](https://www.ncbi.nlm.nih.gov/protein/WP_004525344.1) | **+** | **+** | **+** | **+** | **+** |
| 30 | virA | DP58_RS20365 | [WP_004537674.1](https://www.ncbi.nlm.nih.gov/protein/WP_004537674.1) | **+** | **+** | **+** | **+** | **+** |
| 31 | virG | DP58_RS20370 | [WP_004528788.1](https://www.ncbi.nlm.nih.gov/protein/WP_004528788.1) | **+** | **+** | **+** | **+** | **+** |
| 32 | irlR | DP58_RS22635 | [WP_004197868.1](https://www.ncbi.nlm.nih.gov/protein/WP_004197868.1) | **+** | **+** | **+** | **+** | **+** |
| 33 | boaA | BPS_RS23025 | [WP_080341181.1](https://www.ncbi.nlm.nih.gov/protein/WP_080341181.1) | **+** | **+** | **+** | **+** | **-** |
| 34 | tssI | DP58_RS23075 | [WP_004537132.1](https://www.ncbi.nlm.nih.gov/protein/WP_004537132.1) | **+** | **+** | **+** | **+** | **+** |
| 35 | ahpC | DP58_RS25635 | [WP_004525522.1](https://www.ncbi.nlm.nih.gov/protein/WP_004525522.1) | **+** | **-** | **+** | **+** | **+** |
| 36 | bimA | DP58_RS39755 | [WP_076804876.1](https://www.ncbi.nlm.nih.gov/protein/WP_076804876.1) | **+** | **+** | **+** | **+** | **+** |
