# Supplementary material for: Genome sequence of two novel virulent clinical strains of Burkholderia pseudomallei isolated from acute melioidosis cases imported to Israel from India and Thailand
Source: BMC Genom Data. 2024 May 23;25:47. doi: 10.1186/s12863-024-01225-x (PMC11118722; doi:10.1186/s12863-024-01225-x)
Supplement: Supplementary file 4 — Supplementary Material 4 [file 12863_2024_1225_MOESM4_ESM.pptx]

## Slide 1
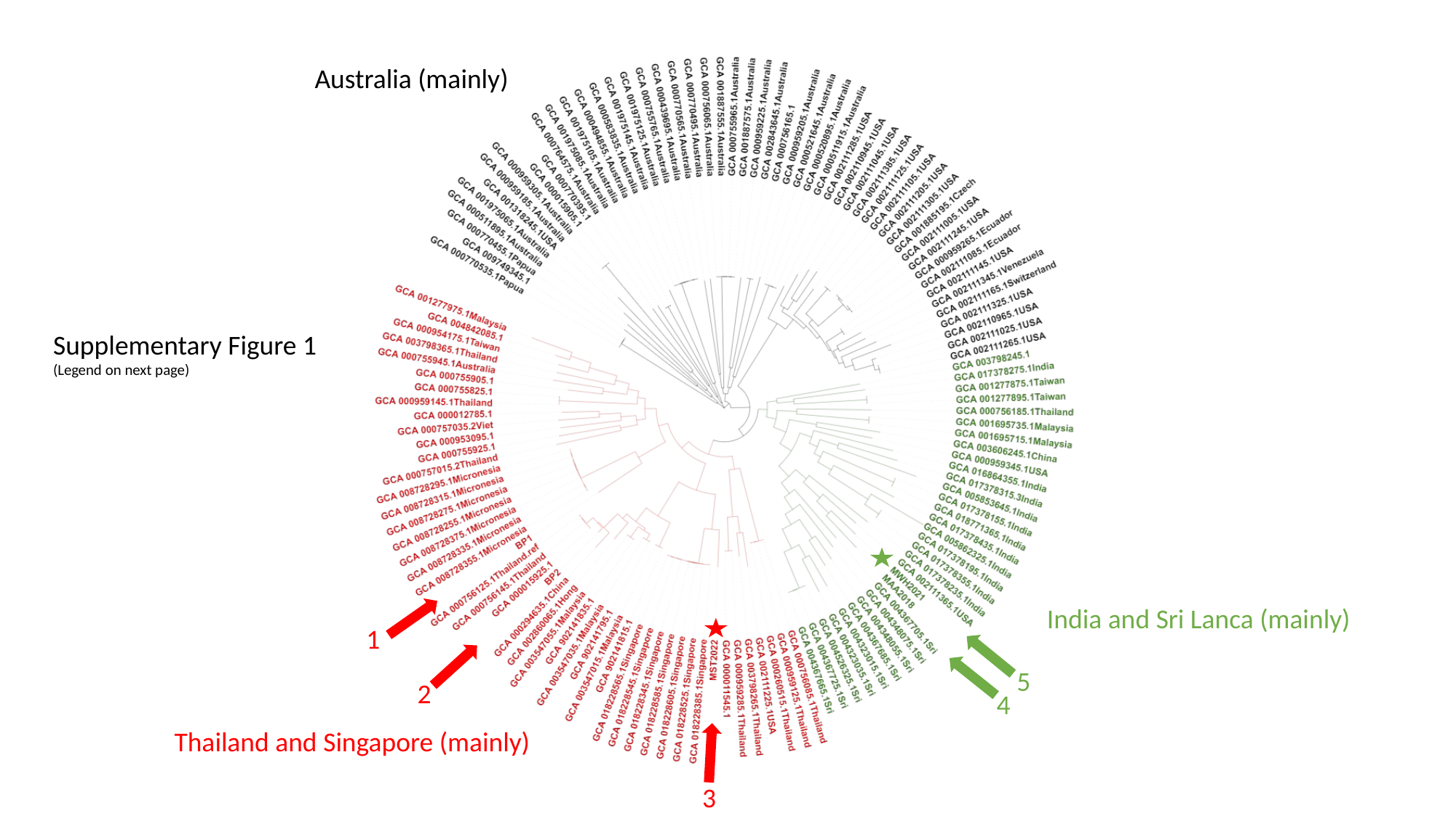

Australia (mainly)
Supplementary Figure 1
(Legend on next page)
India and Sri Lanca (mainly)
1
5
2
4
Thailand and Singapore (mainly)
3

## Slide 2
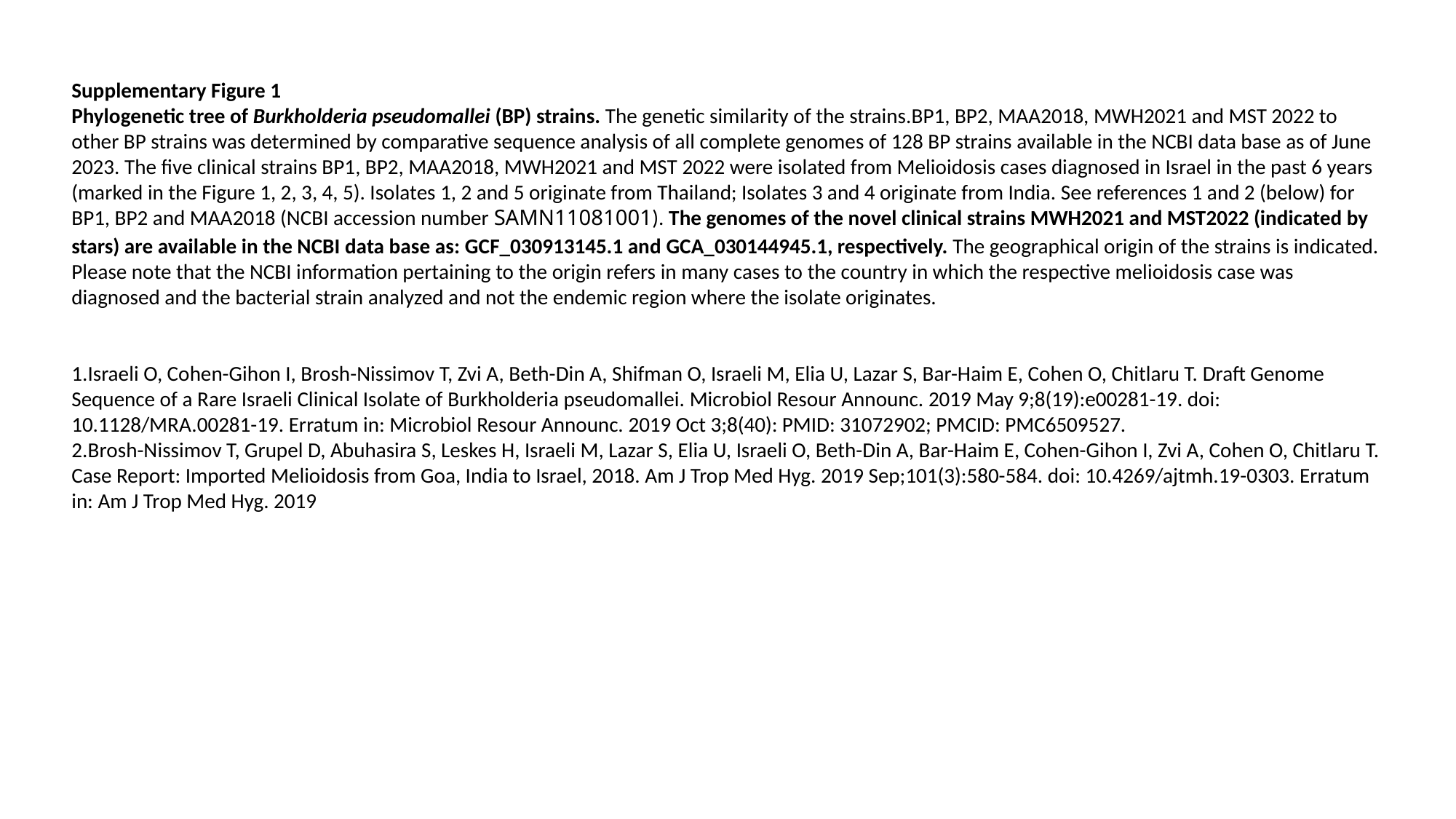

Supplementary Figure 1
Phylogenetic tree of Burkholderia pseudomallei (BP) strains. The genetic similarity of the strains.BP1, BP2, MAA2018, MWH2021 and MST 2022 to other BP strains was determined by comparative sequence analysis of all complete genomes of 128 BP strains available in the NCBI data base as of June 2023. The five clinical strains BP1, BP2, MAA2018, MWH2021 and MST 2022 were isolated from Melioidosis cases diagnosed in Israel in the past 6 years (marked in the Figure 1, 2, 3, 4, 5). Isolates 1, 2 and 5 originate from Thailand; Isolates 3 and 4 originate from India. See references 1 and 2 (below) for BP1, BP2 and MAA2018 (NCBI accession number SAMN11081001). The genomes of the novel clinical strains MWH2021 and MST2022 (indicated by stars) are available in the NCBI data base as: GCF_030913145.1 and GCA_030144945.1, respectively. The geographical origin of the strains is indicated. Please note that the NCBI information pertaining to the origin refers in many cases to the country in which the respective melioidosis case was diagnosed and the bacterial strain analyzed and not the endemic region where the isolate originates.
Israeli O, Cohen-Gihon I, Brosh-Nissimov T, Zvi A, Beth-Din A, Shifman O, Israeli M, Elia U, Lazar S, Bar-Haim E, Cohen O, Chitlaru T. Draft Genome Sequence of a Rare Israeli Clinical Isolate of Burkholderia pseudomallei. Microbiol Resour Announc. 2019 May 9;8(19):e00281-19. doi: 10.1128/MRA.00281-19. Erratum in: Microbiol Resour Announc. 2019 Oct 3;8(40): PMID: 31072902; PMCID: PMC6509527.
Brosh-Nissimov T, Grupel D, Abuhasira S, Leskes H, Israeli M, Lazar S, Elia U, Israeli O, Beth-Din A, Bar-Haim E, Cohen-Gihon I, Zvi A, Cohen O, Chitlaru T. Case Report: Imported Melioidosis from Goa, India to Israel, 2018. Am J Trop Med Hyg. 2019 Sep;101(3):580-584. doi: 10.4269/ajtmh.19-0303. Erratum in: Am J Trop Med Hyg. 2019
